# Healthcare Providers’ Adherence to Recommended Pneumococcal and Influenza Vaccination in Patients Discharged with Respiratory Diseases from General Medical Wards

**DOI:** 10.3390/vaccines11020431

**Published:** 2023-02-13

**Authors:** Amani Alshehri, Marwa Ahmed, Doaa Bagazi, Ahmad Alghamdi

**Affiliations:** 1 Pharmaceutical Care Services, King Saud Medical City, Riyadh 12746, Saudi Arabia; 2 Internal Medicine Department, King Saud Medical City, Riyadh 12746, Saudi Arabia

**Keywords:** descriptive analysis, observational study, influenza vaccine, pneumococcal vaccine, respiratory diseases

## Abstract

The periodic assessment of adherence to vaccination recommendations is an essential component of any vaccination process. This study aimed to investigate the adherence of healthcare providers to the international recommendations on influenza and pneumococcal vaccine in patients discharged from the internal medicine department with respiratory diseases. All medical records of adult patients who are 18 years of age and older with respiratory illnesses and who were discharged in January-February, May-June, and October-November of 2018 were retrospectively analyzed. A total of 264 discharge summaries from 190 patients were included in this analysis. The mean age was 55.5 years, with 54.7% of them being males. Pneumonia was the leading cause of hospitalization (63.7%), followed by asthma or chronic obstructive pulmonary disease (COPD) exacerbation (27.4%). None of the discharged patients had immunization recommendations at discharge or a documented immunization request within 6 months of discharge from the hospital. The findings of this study demonstrated that healthcare providers need to pay more attention to adhering to the global recommendations on influenza and pneumococcal vaccine among patient who were recently discharged with respiratory disease. Additional measures are needed to improve adherence to routinely recommended vaccines among adults with respiratory diseases.

## 1. Introduction

Vaccines are regarded as one of the most effective preventative measures in modern medicine. Almost all people, regardless of age, need immunizations to protect against common infections. The Advisory Committee on Immunization Practices (ACIP) and the Centers for Disease Control and Prevention (CDC) both recommend that age-appropriate vaccines be administered to all individuals, unless there are contraindications [1,2]. Compared with unvaccinated individuals, influenza-vaccinated individuals have a 41–53% [3,4] and 59% reduction in the risk of hospitalization due to influenza and death or intensive care unit (ICU) admissions due to pneumonia, respectively [5]. Similarly, polysaccharide pneumococcal vaccine reduced the risk of death or ICU admissions due to pneumonia by 38% [5].

The periodic assessment of practice performance to recommended vaccines is an essential component of any vaccination process. Assessing the adherence to the recommended vaccines has the advantage of measuring adherence to standards of care, identifying barriers to vaccination, developing strategies for improving vaccination adherence, and optimizing vaccine delivery to targeted patients [6].

To the best of our knowledge, nationwide data are limited on the immunization rates of routine vaccines among Saudi adults [7]. The majority of the officially published data are for children [7,8,9]. In a cross-sectional survey, 44.5% of adult Saudi citizens were vaccinated against seasonal influenza [10]. According to a review of eight cross-sectional studies published between 1989 and 2015, the prevalence of influenza vaccine coverage among the elderly population ranged from 58–73% [11]. Assessment of adult vaccination rates are also limited in other countries, but there is a trend of vaccine underutilization [12,13,14].

The CDC and ACIP both recommend that all children younger than 5 years old should receive the pneumococcal vaccine as part of the routinely recommended vaccines, if not contraindicated. For individuals older than 5 years of age with certain medical conditions, and adults who are 65 years of age and older re-vaccination may be indicated [15]. The annual influenza vaccine is recommended for all individuals who are 6 months of age or older, unless contraindicated [16].

Administering recommended vaccines for eligible patients prior to hospital discharge has been proven to improve vaccination rates among vaccine-eligible individuals [17]. Vaccination among hospitalized individuals has not been associated with increased negative outcomes; such as increased risk of infections, hospital re-admissions, or emergency department visits [18].

### Research Objectives

The primary objective of this study was to assess healthcare providers’ adherence to the global recommendations on influenza and pneumococcal vaccines among patients with respiratory diseases who had recently been discharged from the internal medicine department of a Saudi tertiary hospital. Our secondary objectives were to analyze the number of admissions during the indexed period, length of hospital stay, healthcare providers’ review of immunization history and antibiotic prescription at discharge.

## 2. Materials and Methods

### 2.1. Study Design and Clinical Settings

This was a single-center, observational, retrospective, chart review study. All medical records, discharge summaries and electronic prescriptions of adult patients aged 18 years of age and older, who were discharged with respiratory illnesses in January–February, May–June, and October–November of 2018 were analyzed. The year 2018 was chosen to accurately assess healthcare providers’ adherence to the CDC and ACIP vaccination recommendations prior to the start of the COVID-19 global pandemic. Six months were randomly chosen across the year to minimize the seasonality of some respiratory ailments and infections, which may influence healthcare provider decision making in vaccine prescribing. Patients with incomplete or missing discharge summaries were excluded from this study. Patients were also excluded from this study if they have immunocompromising conditions such as human immunodeficiency virus (HIV) or receiving a high steroid dose for >2 weeks (equivalent to 20 mg of oral prednisone). The study design and process of chart review were supervised and approved by the institutional review board of King Saud Medical City (KSMC), reference number H1RI-05-May19-04.

KSMC is located at the center of Riyadh, the capital city of Saudi Arabia. It is the largest tertiary care training center under the Saudi Ministry of Health (MOH). It has around 1500-bed capacity, and it is recognized as a major referral center for various specialities. For example, intensive care units (ICU), emergency department, cardiology, internal medicine, orthopedics, neurology, oncology/hematology, nephrology, rheumatology, general surgery, pediatrics, maternity services and more.

### 2.2. Data Sources and Collection

The cases were identified using the International Statistical Classification of Diseases, 10th revision, coding system. Chronic obstructive pulmonary disease (COPD), asthma, interstitial lung disease, and upper or lower respiratory tract infections were all considered respiratory diseases in this study. Diagnosis was confirmed on the basis of physician’s notes, X-ray reports, and microbiological results as appropriate.

Respiratory vaccines included the vaccines approved in Saudi Arabia for pneumonia and influenza prevention; the pneumococcal conjugate vaccine (PCV13), the pneumococcal polysaccharide vaccine (PPSV23), as well as the seasonal influenza vaccine. The CDC and ACIP vaccination recommendations were adopted as a reference since they are internationally recognized by most healthcare providers in Saudi Arabia, and they are aligned with vaccination recommendations of most international guidelines [19].

Adherence to the CDC and ACIP vaccination recommendations in patients with respiratory diseases was analyzed by evaluating patients’ electronic discharge summary records, medication history, electronic prescriptions generated in the inpatient or outpatient settings, vaccination certificate in the medical record, and history of immunization prior to hospital admission, if available. These records were retrospectively reviewed by three independent clinical pharmacists for vaccine recommendations, vaccine administration history, antibiotic prescriptions at discharge, as well as vaccine prescriptions issued prior to hospital admission or within six months of hospital discharge. Patients were rated as not adherent to vaccine recommendations if they did not have vaccination certificate included in their medical record, have no vaccination recommendations in the discharge summaries, have no vaccine prescription generated within 6 months of hospital discharge. The length of hospital stay was presented as the average number of days the patients spent in hospital during each hospitalization episode.

### 2.3. Statistics

Descriptive analysis was used for baseline characteristics, as well as primary and secondary objectives. Statistics of continuous variables in demographic and baseline characteristics were summarized as frequency, percentage, mean (standard deviation), and median (interquartile range). All statistical analyses were performed with Microsoft Excel (version 2016, Microsoft, Redmond, WA, USA).

## 3. Results

A total of 264 discharge summaries from 190 patients were included in the analysis. The mean age of the participants was 55.5 ± 21.32 years, with (54.7%, *n* = 104) of them being males. Diabetes (59.4%, *n* = 113), hypertension (41.5%, *n* = 79), and ischemic heart diseases (14.2%, *n* = 27) were the most common comorbidities, as shown in Table 1.

Approximately two-thirds of patients had an underlying lung disease (71%, *n* = 135), such as COPD, asthma, and interstitial lung disease. During the indexed period, (25.3%, *n* = 48) of patients were admitted to the hospital more than twice because of respiratory illness, with pneumonia being the leading cause of hospitalization (63.7%, *n* = 121), followed by asthma exacerbation or COPD exacerbation (27.4%, *n* = 52). The average length of hospital stay was 12 ± 23.1 days. More than half of the patients (53.7%, *n* = 102) were discharged with an antibiotic prescription, and none had documented immunization recommendations at discharge, immunization history prior to hospital admission or immunization administration within 6 months of discharge date.

## 4. Discussion

The study’s findings demonstrate that healthcare providers need to pay more attention to adhering to the recommended vaccinations among patients with respiratory diseases. This was reflected in the lack of immunization recommendations at discharge and absence of a documented immunization, prescribing or administration within 6 months of discharge.

The lack of evidence for adhering to the recommended pneumococcal and influenza vaccines in any of the inspected electronic medical records could be due to various factors. For instance, there are no local policies or institutional immunization protocols to guide physicians in identifying vaccine-eligible patients. There is no designated electronic documentation platform to aid in assessing vaccination status for routinely recommended vaccines, and lastly there is no electronic reminder system integrated with the current electronic health record system to help healthcare providers identify unvaccinated individuals.

The immunization adherence to recommended vaccines among adults in Saudi Arabia can be significantly improved in several ways. First, a national platform should be established to help track adult immunization status and to provide an electronic record for all vaccines administered in any authorized health facility across the country. Second, for vaccine-eligible individuals, proof of vaccination for routine vaccines should be requested prior to college acceptance or hiring. Third, the scope of vaccination awareness campaigns should be broadened by promoting all recommended routine adult vaccines. Fourth, healthcare providers should be educated on the efficacy, safety, indications, and contraindications of vaccines as a preventative healthcare tool. Fifth, any authorized healthcare facility (primary, secondary, tertiary health centers, and community pharmacies) should have clear immunization policies and regular staff training on vaccine administration. Sixth, an electronic reminder system for missed vaccinations should be used. Seventh, the administration of routine immunizations should be scheduled alongside other follow-up visits to reduce frequent commutes to healthcare facilities. Finally, interruptions in vaccine supply should be avoided as they may have an influence on vaccine coverage.

Additionally, other researchers have discussed the underutilization of routinely recommended respiratory vaccines [12,13,14]. A cross-sectional study by Bloom et al. reported that only 1.96% of patients eligible for the influenza vaccine were vaccinated before being discharged from the hospital [14]. Conversely, a prospective observational study by Johnstone et al. demonstrated that only 9% of patients who were eligible for PPSV23 were vaccinated at the time of hospital discharge [5]. Adults discharged with respiratory conditions may be under vaccinated because of system-, healthcare provider-, or patient-related factors [20]. Physicians may choose to postpone vaccination during hospitalization or shortly after discharge if they are concerned that vaccine adverse reactions will lengthen the hospital stay [18]. In addition, fear of side effects is a barrier for patients to receiving a vaccine during hospitalization [18].

Implementing a tool to administer recommended vaccines for eligible patients prior to hospital discharge has been proven to improve vaccination rates among vaccine-eligible individuals [17]. In a quality improvement study by Orenstein, et al., establishing an automatic influenza vaccine order set together with patient education prior to hospital discharge increased the odds of receiving the influenza vaccine by 3.25 times in the intervention group compared to the historical controls [17].

### Study Limitations

This study has limited generalizability because it only included immunocompetent patients discharged from the internal medicine wards at one tertiary health center. Another limitation is the relatively small sample size, which was due to technical difficulties in retrieving discharge summaries for all discharged patients at the specified index period. Moreover, this study did not include all patients who were admitted across the whole year, only six months. This was due to the technical difficulty in individually inspecting all electronic medical records of admitted patients. The authors try to overcome this limitation by randomly selecting different months across the different seasons of the year to give an overall picture of the healthcare providers’ adherence to vaccination recommendations. These limitations must be considered when interpreting the findings of this study, as they may not reflect healthcare providers’ awareness of patients’ immunization status in all clinical settings (e.g., long-term facilities or ambulatory care clinics) or across different specialties (e.g., pediatrics, hematology, oncology, or rheumatology, or patients with immunocompromising conditions).

## 5. Conclusions

In conclusion, this study assessed the adherence of healthcare providers to the recommendations on pneumococcal and influenza vaccination among patients with respiratory diseases who had recently been discharged from the internal medicine department of a Saudi tertiary hospital. The findings of our study are alarming, as they place a burden on the healthcare system to improve adherence to vaccine recommendations among adults with respiratory illnesses. These findings highlight the need for developing additional measures to improve adherence to vaccine recommendations. Local and national guidelines and policies on vaccine administration among high-risk individuals are needed. Data from different specialties and other healthcare settings are required to reflect healthcare providers’ adherence to vaccine recommendations across Saudi Arabia.

## Figures and Tables

**Table 1:** Baseline characteristics.

Total Number of Patients (N = 190).	
**Age, years**	
Mean ± SD	55.5 ± 21.32
Median (IQR)	56.05 (38)
**Male sex,** ***n*** **(%)**	104 (54.7)
**Preexisting comorbidities**	
Ischemic heart diseases, *n* (%)	27 (14.2)
Hypertension, *n* (%)	79 (41.5)
Diabetes mellitus, *n* (%)	113 (59.4)
Underlying lung diseases, *n* (%)	135 (71)
**Diagnosis on admission**	
Pneumonia, *n* (%)	121 (63.7)
Asthma and COPD exacerbation, *n* (%)	52 (27.4)
Other respiratory infections, *n* (%)	17 (8.9)
**Number of admissions per index period, Mean ± SD**	1 ± 0.93
**Patient admitted twice or more during the indexed period, ** ***n*** **(%)**	48 (25.3)
**Length of hospital stay, days**	
Mean ± SD	12 ± 23.1
Median (IQR)	7 (7)
**Antibiotics prescribing at discharge, ** ***n*** **(%)**	102 (53.7)
**Immunizations recommended at discharge, ** ***n*** **(%)**	0 (0)
**Documented immunization history prior to hospital admission or within 6 months of discharge, ** ***n*** **(%)**	0 (0)

SD: standard deviation. IQR: Interquartile range. Underlying lung diseases include (COPD, asthma, interstitial lung disease, or structural lung diseases). COPD: chronic obstructive pulmonary disease. Other respiratory infections include (viral and atypical respiratory tract infections). Index period (was the hospital discharges in January to February, May to June, and October to November of 2018).

## Data Availability

All data available upon request.

## Abbreviations

The following abbreviations are used in this manuscript:

ACIP	Advisory Committee on Immunization Practices
CDC	Center for Disease Control and Prevention
ICU	Intensive Care Unit
COVID-19	Coronavirus Disease of 2019
KSMC	King Saud Medical City
MOH	Ministry of Health
HIV	Human Immunodeficiency Virus
COPD	Chronic obstructive pulmonary disease
PCV13	Pneumococcal Conjugate Vaccine
PPSV23	Pneumococcal Polysaccharide Vaccine
